# Acute Detection of VENtricular Thrombus by Technologists (ADVENTT study) and the impact of an image interpretation teaching intervention

**DOI:** 10.1186/1532-429X-18-S1-T1

**Published:** 2016-01-27

**Authors:** Chris B Lawton, Jonathan C Rodrigues, Amardeep Dastidar, Chiara Bucciarelli-Ducci

**Affiliations:** grid.410421.20000000403807336Cardiac MRI Unit, Bristol Heart Institute, Bristol, United Kingdom

## Background

The detection of a ventricular thrombus requires immediate anti-coagulation to prevent the risk for stroke. Prompt diagnosis is of pivotal importance. Excluding a ventricular thrombus is part of the routine assessment of reporting viability studies. We aimed to determine the detection rate of ventricular thrombus by CMR technologists before and after a teaching intervention by CMR doctors.

## Methods

A total of 2,481 CMR studies performed in the Bristol Heart Institute CMR Unit between Jan - Dec 2015 were reviewed. A cohort of 25 consecutive patients with ischemic cardiomyopathy were identified,10 patients with, and 15 patients without LV thrombus. A multi-parametric CMR protocol was performed in all patients which included cines, early gadolinium enhancement (EGE) and late gadolinium (LGE) sequences, acquired in both the long-axis and short-axis planes. All scans were reported by a consultant with >10 years' experience in CMR. Seven technologists independently reviewed the 25 anonymised and randomised studies on a dedicated workstation (cvi42, Circle Cardiovascular Imaging) and documented the presence or absence of thrombus, and their confidence level on a 7-point Likert scale (1 least confident, 7-most confident). Two CMR senior fellows (> 3 years of experience) delivered a focussed teaching programme to the 7 technicians. The assessment of all 25 randomised scans was repeated after the teaching intervention (1 month apart from the baseline assessment) Statistical analysis was performed with paired T-tests. Significance was set at two-tailed P < 0.05.

## Results

The overall technologists' ability to correctly identify and exclude ventricular thrombus at baseline was 78 ± 5%. Following dedicated training, there was an improvement in the ability of detecting thrombus, albeit non significant (pre-training: 78 ± 5 vs post-training: 83 ± 9%, P = 0.172). Out of the 7 technologists, 5 showed a significant improvement in diagnosing thrombus (79 ± 5 vs 88 ± 4 Post teaching, P0.005).There was no significant difference in the diagnostic performance of the other 2 radiographers (76 ± 0 vs 70 ± 3 p = 0.205) who recorded a worse diagnostic performance in detecting thrombus after the teaching intervention. However, there was a significant improvement both in the ability (Pre: 74 ± 13 vs Post: 82 ± 11%, P<0.05) and in the confidence (Pre: 5.2 ± 0.9 vs Post: 5.7 ± 0.8, p < 0.05) to exclude thrombus following training (Figure 2).

**Figure 1:**
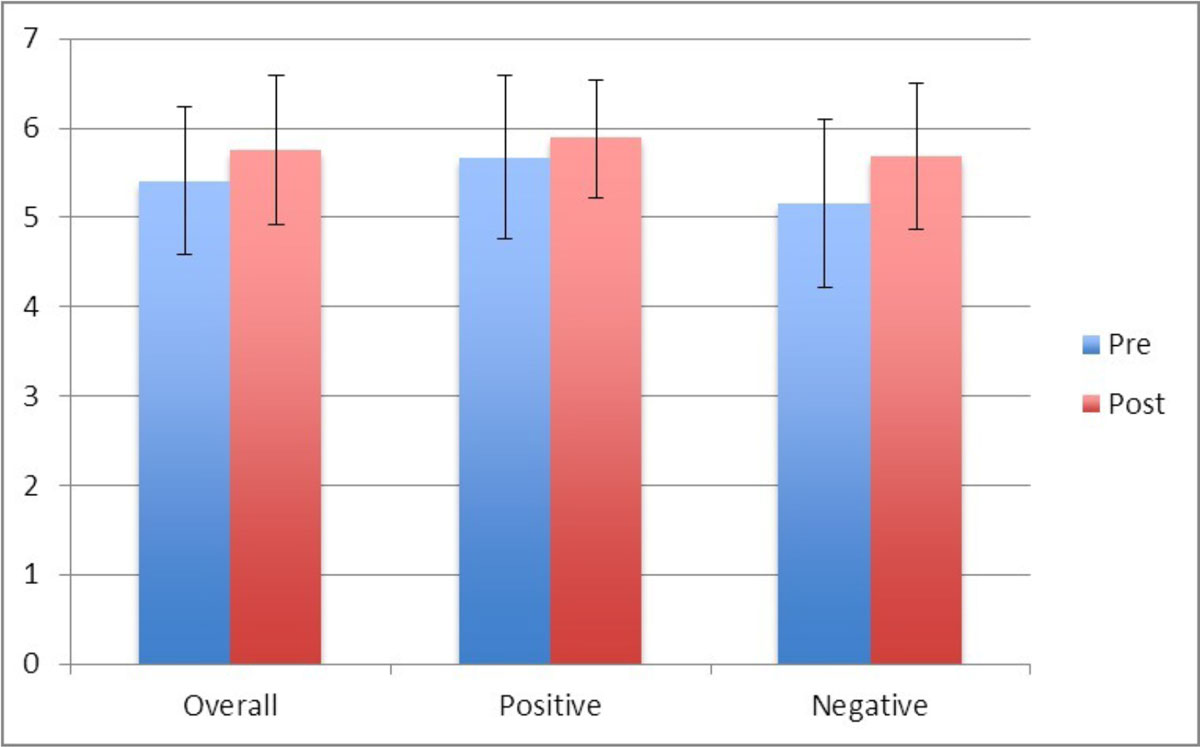
**A graph showing impact of training on Technologists confidence at detecting and excluding ventricular thrombus**. *P <0.05

**Figure 2:**
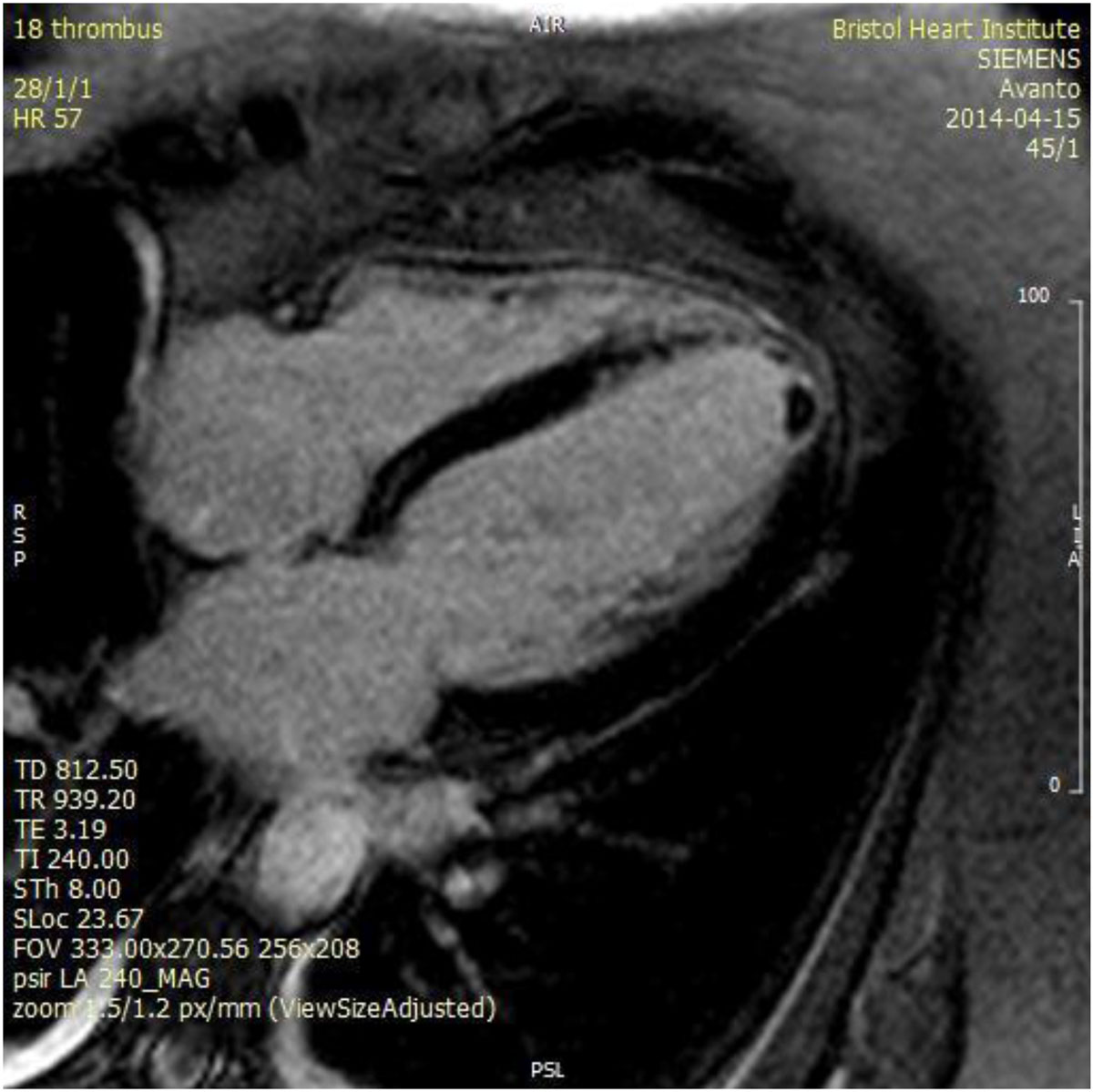
**Long axis post gadolinium image showing apical thrombus**.

## Conclusions

This is the first study exploring the role of CMR technologists in detecting LV thrombus in clinical viability scans, and demonstrates they can identify and exclude life-threatening LV thrombus to a high standard. It is also the first study to explore the impact of a training intervention to improve the technologists' diagnostic ability to detect thrombus, which improved after the teaching intervention offered by the doctors. The role of the technologist in routinely screening for LV thrombus during the image acquisition of a viability study should be further explored in larger studies.

